# Primary Signet-Ring Cell Carcinoma of the Urinary Bladder Successfully Managed with Radical Cystectomy in a Young Patient

**DOI:** 10.1155/2017/9121078

**Published:** 2017-06-05

**Authors:** Farzad Allameh, Morteza Fallah Karkan, Yalda Nilipour, Azadeh Rakhshan

**Affiliations:** ^1^Urology Department, Shohada-e-Tajrish Hospital, Shahid Beheshti University of Medical Sciences, Tehran, Iran; ^2^Pediatric Pathology Research Center, Mofid Children's Hospital, Shahid Beheshti University of Medical Sciences, Tehran, Iran; ^3^Pathology Department, Shohada-e-Tajrish Hospital, Shahid Beheshti University of Medical Sciences, Tehran, Iran

## Abstract

Primary signet-ring cell adenocarcinoma of bladder is a rare neoplasm, usually seen in middle age adults. We report the case of an 18-year-old man who presented with intermittent gross hematuria. Computed tomography imaging showed multifilling defects in the bladder. The patient underwent a transurethral resection of the bladder tumor. Histological findings were consistent with poorly differentiated mixed mucinous and signet-ring cell adenocarcinoma. We ruled out other possible origins of tumor by gastrointestinal endoscopy and colonoscopy. The patient was treated with radical cystectomy with prostate and seminal vesicle sparing technique and orthotopic diversion using “W” ileum pouch with pelvic lymphadenectomy to the bifurcation of the aorta was done. Six-month follow-up of patient showed normal conditions without metastatic spread or any recurrence.

## 1. Introduction

Primary signet-ring cell carcinoma (PSRCC) of the urinary bladder, which was first reported by Saphir in 1955 [1, 2], is a rare variant of adenocarcinoma and comprises only 0.24% to 2% of all primary epithelial urinary bladder tumors. There should be a rationale cause for producing adenocarcinoma in an organ without normal glandular tissue. There are two acceptable hypotheses to explain that: first one is the metaplastic change of the normal urothelium to a mucinous or glandular epithelium and the second is the embryologic persistence of endodermal intestinal tissue [3].

We report a case that was treated with radical cystectomy using prostate and seminal vesicle sparing technique. We created an orthotopic diversion with “W” ileum pouch for him.

## 2. Case Presentation

An 18-year-old Afghan man with a history of cystolithotomy seven years ago in Kabul, Afghanistan, was admitted to Shohada-e-Tajrish Hospital with the complaint of 1-year intermittent total gross painless hematuria with amorphous clots. There was no conclusive history from the patient, and no medical records could be obtained documenting such evidence. The medical and familial history were unremarkable.

**Figure 1:**
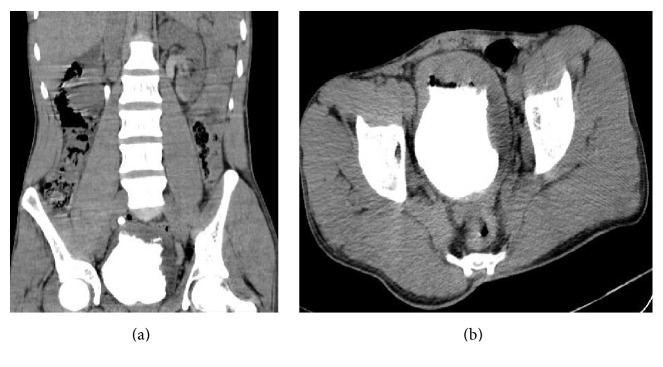
Abdominopelvic CT-scan of patient. (a) Axial view showing bilateral hydronephrosis and nonuniform decreased cortex; (b) horizontal view showing multiple filling defects in bladder.

Ultrasound evaluations revealed nonuniform decrease in both kidney parenchyma and bilateral hydronephrosis which was dominant in the left side (Grade III) and partial defect on the left bladder wall. Computed tomography (CT) imaging with and without intravenous contrast administration demonstrated bilateral hydronephrosis which was dominant in the left side; nonuniform decrease in the cortex of both kidneys and multiple filling defects were present in the bladder lumen without distant metastasis (Figure 1).

After clinical examination, the patient underwent a transurethral resection of the bladder tumor (TURT) that revealed a white, calcified sessile tumor extending from the left bladder wall to the dome and obstructing the left ureteral orifice. Tissues were taken by bipolar loop resection and left nephrostomy was inserted. Specimen was sent for the polymerase chain reaction to evaluate tuberculosis, because of race or ethnicity (Afghanistan) where TB is widespread, diffuse uretral stricture, nonpapillary mass, and diffuse erythema; and the results were negative.

Histological findings were consistent with poorly differentiated mixed mucinous and signet-ring cell adenocarcinoma. Immunohistochemical (IHC) studies demonstrated strong positivity for CK7, CK20, and CDX-2 and negativity for PSA, CK34*β*E12, and Vimentin. Primary urinary bladder adenocarcinoma versus metastatic adenocarcinoma from gastrointestinal tract origin was suggested.

After this pathology report, hematology and radiotherapy consults suggest gastrointestinal tract workup. Esophagogastroduodenal endoscopy, rectosigmoidoscopy, and analysis of tumor markers CA 19-9 and CA 125 were performed to exclude other possible extravesical primary lesions. Transrectal needle biopsy of prostate was done and all examinations showed normal results.

After this diagnosis, the patient underwent radical cystectomy with prostate and seminal vesicle sparing technique and orthotopic diversion with “W” ileum pouch made for him, pelvic lymphadenectomy to the bifurcation of the aorta was performed, and bilateral JJ catheters were embedded in both ureters. We chose this technique because the patient was single and emphasized his future potency.

**Figure 2:**
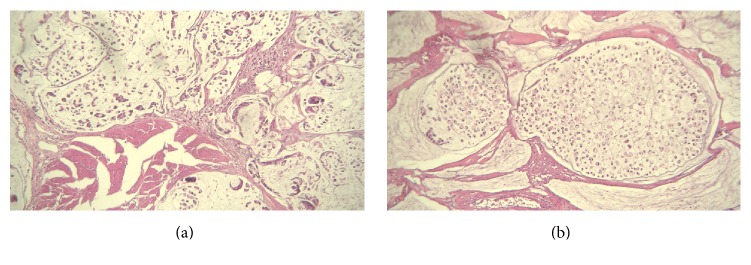
(a and b) Hematoxylin and Eosin stain: tumor was mainly composed of mucin lakes containing cords and nests of the tumor cells and some lakes bearing only signet-ring cells. Original magnification: ×200 in (a) and ×400 in (b).

Macroscopic examination of the cystectomy specimen showed an ill-defined ulcerative and necrotic tumor mass with soft and gelatinous consistency filling the whole bladder cavity measuring 2.5 cm in maximal thickness. Microscopically, the bladder wall was infiltrated into deep muscularis propria (outer half) by a necrotic neoplasm composed of lakes of mucins containing floating nests or cords of epithelioid cells and many signet-ring cells (Figures 2(a) and 2(b)). No urachal remnant was identified. The diagnosis of signet-ring variant of mucinous adenocarcinoma of the urinary bladder was made. No metastatic lymph node was identified (T2N0M0).

Six months postoperatively, clinical examinations, chest X-rays, CT scans, and cystoscopy showed normal results without distal spread or any recurrence.

## 3. Discussion

PSRCC of the bladder is a rare type of adenocarcinoma of the bladder. Less than 100 cases have been reported in the literature [4]. Most of the patients were in their seventh to eighth decades of life and there was male predominance and they were usually diagnosed at an advanced stage, usually demonstrating a subsequently poor prognosis [4]. In the present study, the patient developed signet-ring cell adenocarcinoma in the second decade which was unique in the literature.

Primary adenocarcinoma of the urinary bladder is derived from urothelium that underwent glandular metaplasia, often in the context of chronic irritation of the vesical mucosa. Metaplasia of cystitis cystica by mucin-producing columnar cells leads to cystitis glandularis which was considered the precursor lesion of primary adenocarcinoma. This glandular metaplasia may also occur without urothelial invagination, resulting in a variable cystoscopic appearance: sessile, papillary, nodular, or ulcerated. The intestinal, clear cell, and signet-ring cell subtypes are the best known subtypes in the literature [5]. In our case presentation, the mass was sessile and in pathology evaluation it was signet-ring type. Columnar metaplasia of epithelial surface happens with no downward invagination. Predictor variables of such alterations are chronic vesical irritation and infection [1]. This can be the predisposing factor in the case reported in the present study who underwent cystolithotomy when he was 11 years old. Despite such neoplasms nature in secreting of mucin, mucus existence in urine is very rare. Interstitial part is the most frequent place for mucus deposition. In seldom cases, mucin is passed through the lumen in acini and its excess changes the nuclei to peripheral crescent and makes a signet-ring shape [5].

Clinical presentation of PSRCC of the bladder is similar to other bladder malignancies and hematuria is the most common presenting symptom. Another rare presentation in the literature is mucinuria which is reported in 3–12% of the patients [5]. Our patient had history of one-year intermittent painless hematuria with clot as the presenting symptom.

One of the main problems in cases of PSRCC of the bladder is to exclude metastatic adenocarcinoma from other sites of the body. As secondary adenocarcinoma is more common than primary adenocarcinoma and differentiating the two in cytologic preparations can be difficult [5], we evaluated the other possible secondary origin of this tumor in our patient at a young age by gastroduodenoscopy and rectosigmoidoscopy. Another tumor that can invade the bladder by local extension and mimic primary bladder adenocarcinoma is prostate adenocarcinoma [6] which was ruled out by transrectal needle biopsy of prostate.

Though the majority of tumors occur near the trigone, primary adenocarcinoma may arise anywhere in the bladder and may be either unifocal or multifocal. Treatment is surgical and consists of radical cystectomy with pelvic lymph node dissection. Chemotherapy is of questionable value [7]. Treatment modalities of PSRCC of the bladder are surgery, radiotherapy, and chemotherapy. Surgical options are transurethral resection, partial cystectomy (for urachal lesion or tumor in diverticulum), and radical cystectomy with urinary diversion. Unfortunately, no standard chemotherapy exists for PSRCCs of the bladder because of their rarity [5].

Fujita et al. [8] reported a 60-year-old case of signet-ring cell carcinoma of the urinary bladder successfully treated with radical cystectomy and creation of an ileal neobladder. The histopathological stage was pT3aN0M0 and adjuvant chemotherapy (TS-1) was performed. Romics et al. [9] presented a case of a 45-year-old woman with gross hematuria showing signet-ring cell carcinoma. They performed radical cystectomy with ureterosigmoidostomy (Mainz pouch II) and the patient received adjuvant chemotherapy with cisplatin and fluorouracil following surgery. Cobo-Dols et al. [1] reported a case of PSRCC treated successfully with total cystectomy followed by systemic chemotherapy with cisplatin and gemcitabine, a standard combination for transitional carcinoma of the urinary bladder. Hirano et al. [10] reported success with primary treatment of signet-ring cell adenocarcinoma of the bladder with intra-arterial chemotherapy. Until this report, neoadjuvant chemotherapy for treatment is not recommended.

According to patient age and the risk of urachal remnant, clinician must consider this diagnosis. Tazi et al. [11] presented three cases of lower abdominal pain with final diagnosis of urachal diseases without bladder wall involvement successfully treated with antibiotic and surgical resection of tract or complicated sinus with bladder cuff. All of pathology examination of cases was negative for bladder malignancy.

## 4. Conclusion

Diagnosis of adenocarcinoma of the urinary bladder should be confirmed by the evaluation of all the primary sites of adenocarcinoma such as the gastrointestinal, lung, and breast sites, before labeling it as a PSRCC of the urinary bladder. Previous reports showed that the prognosis of PSRCC was too poor and, at the time of diagnosis, most patients reached a high stage of disease. However, herein we reported a young patient who, to our knowledge, was the first case of primary signet cell adenocarcinoma of bladder in this age. Performing surgical procedures, as well as chemotherapy and radiotherapy, was not generally successful in the literature and only total cystectomy might offer some hope for patients.
